# Orthotopic Liver Transplantation in Human-Immunodeficiency-Virus-Positive Patients in Germany

**DOI:** 10.1155/2012/197501

**Published:** 2012-07-30

**Authors:** E. Anadol, S. Beckebaum, K. Radecke, A. Paul, A. Zoufaly, M. Bickel, F. Hitzenbichler, T. Ganten, J. Kittner, M. Stoll, C. Berg, S. Manekeller, J. C. Kalff, T. Sauerbruch, J. K. Rockstroh, U. Spengler

**Affiliations:** ^1^Department of Internal Medicine I, University of Bonn, 53105 Bonn, Germany; ^2^Department of General, Visceral and Transplantation Medicine, University Hospital Essen, 45122 Essen, Germany; ^3^Department of General, Visceral and Transplantation Surgery, University Hospital Essen, 45122 Essen, Germany; ^4^Department of Internal Medicine, University of Hamburg, 20251 Hamburg, Germany; ^5^Department of Internal Medicine, Frankfurt University, Hospital Medical Centre, 60590 Frankfurt, Germany; ^6^Department of Internal Medicine I, University of Regensburg, 93042 Regensburg, Germany; ^7^Department of Internal Medicine, Heidelberg University, 69125 Heidelberg, Germany; ^8^Department of Internal Medicine, Mainz University, 55122 Mainz, Germany; ^9^Department of Immunology and Rheumatology, Medical University of Hannover, 30625 Hannover, Germany; ^10^Department of Internal Medicine, Tübingen University Medical School, 72076 Tübingen, Germany; ^11^Department of Surgery, University of Bonn, 53105 Bonn, Germany

## Abstract

*Objectives*. This summary evaluates the outcomes of orthotopic liver transplantation (OLT) of HIV-positive patients in Germany. *Methods*. Retrospective chart analysis of HIV-positive patients, who had been liver-transplanted in Germany between July 1997 and July 2011. *Results*. 38 transplantations were performed in 32 patients at 9 German transplant centres. The reasons for OLT were end-stage liver disease (ESLD) and/or liver failure due to hepatitis C (HCV) (*n* = 19), hepatitis B (HBV) (*n* = 10), multiple viral infections of the liver (*n* = 2) and Budd-Chiari-Syndrome. In July 2011 19/32 (60%) of the transplanted patients were still alive with a median survival of 61 months (IQR (interquartile range): 41–86 months). 6 patients had died in the early post-transplantation period from septicaemia (*n* = 4), primary graft dysfunction (*n* = 1), and intrathoracal hemorrhage (*n* = 1). Later on 7 patients had died from septicaemia (*n* = 2), delayed graft failure (*n* = 2), recurrent HCC (*n* = 2), and renal failure (*n* = 1). Recurrent HBV infection was efficiently prevented in 11/12 patients; HCV reinfection occurred in all patients and contributed considerably to the overall mortality. *Conclusions*. Overall OLT is a feasible approach in HIV-infected patients with acceptable survival rates in Germany. Reinfection with HCV still remains a major clinical challenge in HIV/HCV coinfection after OLT.

## 1. Introduction

The introduction of highly active antiretroviral therapy (HAART) in 1996 has enabled the control of human-immunodeficiency-virus (HIV)-infection in most patients and resulted in a marked decrease in opportunistic infections and an increase in life expectancy [1, 2]. Since HIV has now become a chronic disease, comorbidities are of increasing clinical importance. Among HIV-infected patients in Germany HCV coinfection rates range between 10 and 15% [3], and the prevalence of HBV surface antigen (HBs-Ag) is estimated to be 10% [4]. Thus end-stage liver disease has become a prominent problem in these patients, and the demand for liver transplantation is increasing. Indeed, liver-associated mortality of HIV-coinfected subjects with viral hepatitis has become a leading cause of death in many countries [5, 6]. Despite recent advances in the treatment of chronic hepatitis B and hepatitis C, OLT remains the last resort for patients with decompensated liver cirrhosis. According to the 1993 consensus conference on indications for liver transplantation HIV infection was initially considered a contraindication, but the advent of HAART in 1996 improved prognosis of HIV-infected patients and has encouraged many transplant centers to accept selected HIV-positive candidates, and meanwhile more than 300 liver transplantations have been reported worldwide with overall promising outcomes [7–10]. 

Here we evaluate the outcomes of all liver transplantations in HIV-positive patients receiving a liver graft between July 1997 and July 2011 in Germany.

## 2. Patients and Methods

After contacting all German transplant centers which had offered transplantation to HIV-infected patients, we conducted a retrospective observational cohort study.

 A standardized questionnaire comprising general demographic data (gender, age, and body weight), characteristics of HIV infection (date of diagnosis, CDC stadium, transmission risk factor, CD4 cell count, quantitative HIV-RNA, use of HAART), and details of liver disease and OLT (type of diagnosis, biochemical and clinical signs of liver disease, complications of OLT) was used for collecting data. The data were analyzed for 4 different time points: at the listing for OLT, at OLT itself, at (re)initiation of HAART after OLT and finally at the last available followup. 

The data were evaluated with regard to criteria for patient selection, organ dysfunction, and reasons for OLT and mortality. The Kaplan-Meier method was used to estimate the survival function. In addition to survival, causes of death as well as therapy and recurrence of viral hepatitis were analyzed. The model for end-stage liver disease (MELD) score [11], which has been used for organ allocation since December 2006, was calculated as a marker of liver disease severity.

## 3. Results

### 3.1. Criteria for Patient Selection

All centers reported that criteria for selecting a candidate for OLT were CD4 counts ≥100 cells/*μ*L and HIV-RNA below the limit of detection or at least a reasonable option for efficient HAART if antiviral medications were to be started in the posttransplant period. Indeed, five patients each had a history of an opportunistic infection prior to OLT: varicella-zoster virus infection, esophageal candidiasis, pneumocystis pneumonia, CMV disease, and tuberculosis; the 4 patients with AIDS-defining complications were on effective HAART prior to listing for liver transplantation, and opportunistic diseases had been successfully controlled before OLT. An AIDS-defining event prior to listing of OLT was not considered as an exclusion criterion unless they were chronic diseases with limited treatment options (e.g., progressive multifocal leukoencephalopathy, chronic cryptosporidiosis, and multidrug-resistant systemic fungal infections).

All centers agreed that contraindications for transplantation were ongoing alcohol or drug abuse, advanced HCC metastases in other organs, other concurrent malignant disease, advanced cardiopulmonary disease, and age > 65 years. 

### 3.2. Characterization of Transplanted Patients

In total 38 transplantations were performed in 32 HIV-positive patients, including 2 re-retransplantations and 2 retransplantations. Ten patients were transplanted in Bonn, 8 in Essen, 3 in Heidelberg, 3 each in Hamburg and Frankfurt, 2 in Mainz, and one patient each in Tübingen, Hannover, and Regensburg.

**Figure 1:**
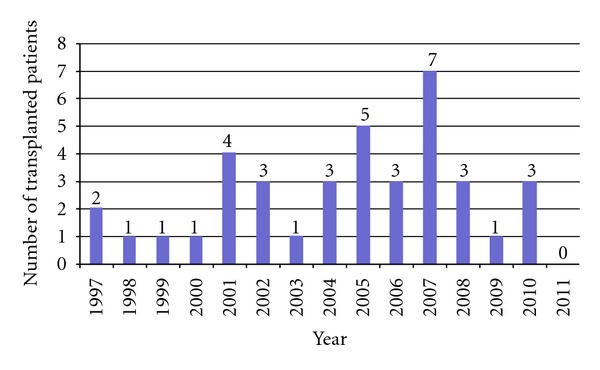
Thirty-eight liver transplantations performed on 32 HIV-infected patients from July 1997–July 2011 in Germany.

Overall, 31 male and 1 female patients were treated by liver transplantation during the observation period, one of whom had also received a kidney graft due to acute kidney failure on a preexisting chronic kidney disease. The median age at transplantation was 47 years (IQR, 37–52), and patients had a median duration of HIV infection of 16 years (IQR, 12–19) (Table 1). The annual rates of liver transplantation in the German HIV-positive patients are shown in Figure 1. Details on immunosuppression were available only from 5 centers (25 patients). Also in 2 HIV-positive patients with recurrent hepatitis C, data concerning antiviral HCV therapy were missing. 

Transplantation was done under the high urgency code for 4 patients with acute liver failure due to a hepatitis B: these received an organ between 1 and 7 days after listing. One patient was listed urgently for combined kidney and liver transplantation to treat acute liver failure after fluconazole and coexisting insufficiency. This patient received a liver and kidney graft 44 days after listing. In elective patients livers were allocated according to waiting time until December 2006, when the MELD system was introduced. 

Median waiting time was 145 days for elective patients listed in the pre-MELD era and 120 days for patients listed after December 2006. Further patient characteristics are summarized in Tables 1 and 2.

Concerning HIV infection, two of the patients with fulminant hepatitis B were incidentally diagnosed to be HIV-positive during workup for OLT. One patient with chronic hepatitis B was discovered to be HIV-positive after OLT. In 29 patients HIV-infection had been known prior to evaluation for liver transplantation. In the 3 remaining patients the temporal relationship between the diagnosis of HIV-infection and evaluation for transplantation was not reported.

At the time of transplantation all patients had CD4 T-cell counts above ≥100/*μ*L; 71% of the patients had CD4 T-cell counts ≥200 cell/*μ*L; HIV-RNA was below the limit of detection of 50 copies/ml in 16 patients (52%). The median MELD score at the day of transplantation was 17 (IQR, 13–29) (*N* = 27). Prior to transplantation ascites had occurred in 14 patients, variceal hemorrhage in 4 patients, and hepatic encephalopathy in 8 patients.

### 3.3. Complications after OLT

Nineteen of the 32 transplanted patients in Germany are still alive in 2011 after a median followup of 61 months (IQR, 41–86) giving rise to an overall mortality of 41%.

In 5 centres where more than 2 patients were transplanted, mortality varied between 20% and 63%. Causes of death are summarized in Table 3.

Six patients died in the early posttransplantation period during the first 3 months after liver transplantation. 

Seven patients died 8, 10, 13, 25, 31, 56, and 93 months after liver transplantation, respectively. 

One patient had received a graft from an HCV-positive donor. This patient suffered recurrent hepatitis C rapidly progressing to cirrhosis and received a second graft 4.5 years after the initial transplantation; he had primary graft dysfunction after retransplantation and died from sepsis 3 months after receiving a second liver graft. Retransplantation, owing to primary graft nonfunction and ischemic type biliary lesions (ITBLs), had been successfully performed in one patient each. Finally, re-retransplantation was successful in a HIV/HBV-coinfected patient who developed decompensated secondary biliary cirrhosis 13 years after his second transplantation. 

Graft rejection was observed in 10 patients, which was lethal in one patient but was successfully treated with high-dose prednisone in the remaining 8 patients. Another patient suffered tonic clonic convulsions requiring transient reintubation during postoperative recovery and ultimately developed left-sided hemiparesis. 

CMV viremia was detected in 5 patients and preemptively treated with intravenous ganciclovir. One patient developed cutaneous Kaposi's sarcoma 126 days after OLT. Reduction of immunosuppression led to a transient regression. However, Kaposi's sarcoma in combination with multicentric Castleman's disease reoccurred 18 months later although CD4 counts were above 350 CD4-cells/*μ*L at this time. This patient was successfully rescued by liposomal doxorubicin, and he is still alive after 96 months. In another patient secondary liver failure occurred after chemotherapy for Hodgkin's disease 17 months after OLT.

### 3.4. HAART and Immunosuppression

Reinitiation of HAART was delayed for a median of 16 days (IQR, 2–31) after OLT in 22/29 patients. Five patients received HAART continuously. Three patients did not receive HAART after OLT due to poor liver function. They died early after 22, 24, and 57 days, respectively. In one patient HAART was initiated after 21 months. This patient spontaneously showed persistently low HIV-RNA after OLT and high CD4 counts above the thresholds for starting HIV therapy at that time; he recently was re-retransplanted 13 years after his second transplantation (see Section 3.3). In two patients data on antiretroviral management were not available. Details of antiretroviral therapy are summarized in Table 1. Azidothymidine (AZT) was used in HAART in 6 and 4 patients before and after OLT, respectively. Also stavudine (D4T) had been given before and after OLT in 6 and 4 patients, respectively. Two of the patients had died from causes unrelated to this drug (patients 2 and 6 in Table 3). In the other two patients D4T was changed to PIs later on.

Concerning immunosuppression detailed information was provided from 5 centers covering 25 patients. Nineteen patients received cyclosporine A (CyA) in addition to prednisone. In 3 patients calcineurin-inhibitor-associated nephrotoxicity was observed, and patients were then continued on mycophenolate mofetil (MMF) and reduced CyA doses. Three received tacrolimus (TAC) in combination with MMF. In 4 patients immunosuppression had been started with CyA and was switched to TAC later on.

### 3.5. Recurrence of Viral Hepatitis

**Figure 2:**
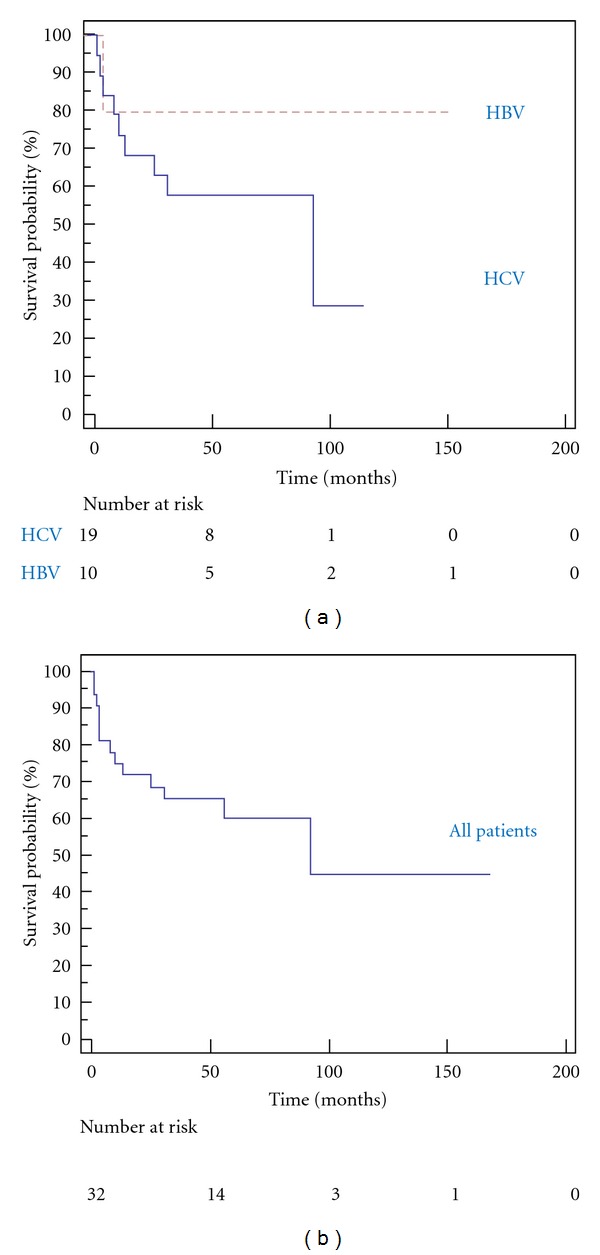
Kaplan-Meier survival after liver transplantation. The left Kaplan-Meier plot illustrates survival probabilities stratified for HIV/HCV and HIV/HBV coinfection. Two patients with HCV/HBV and HCV/HBV/HDV coinfection had been excluded in this analysis. The right plot illustrates survival probabilities for all HIV-positive patients after liver transplantation.

Mortality after OLT was 20% (2/10) in HIV/HBV-coinfected patients and 47% (9/19) in HIV/HCV-coinfected patients. Survival in HIV/HCV- and HIV/HBV-coinfected after liver transplantation is summarized in Figure 2 illustrating a 58% and 80% estimated 5-year Kaplan Meier survival, respectively.

HBs hyperimmunoglobulin and/or HBV-active antiretroviral therapy prevented recurrence of hepatitis B in all but one patient. This patient had received tenofovir (TDV) and lamivudine (3TC) without HBs-hyperimmunoglobulin and developed low-level HBV viremia 1 year after OLT. Details of HBV prophylaxis with HBs-hyperimmunoglobulin in combination with nucleoside or nucleotide analogues are shown in Table 4. 

19 HIV-positive patients were transplanted for chronic hepatitis C. One patient each had HCV/HBV/HDV and HCV/HBV coinfection (Table 1). HCV reinfection occurred in all 21 HIV-HCV coinfected patients after OLT. Of note, one patient spontaneously cleared his acute recurrent hepatitis C in the immediate postoperative period under HAART. 

HCV genotypes were available in 14 patients; HCV genotype 1 and 4 infection was present in 10 (71%) patients and genotype 2 and 3 in 4 (29%) patients. Details of recurrent hepatitis C are summarized in Table 5.

 12 patients did not receive anti-HCV therapy, 7 of whom had died (Table 5), while the other 5 patients are still alive after a median of 36 months (IQR, 28–48).

Therapy with pegylated interferon plus ribavirin (plus amantadine in 3 patients) was attempted in 7 patients after a median of 69 days (IQR, 39–107) after OLT. Five of the 7 patients (72%) achieved a sustained treatment response (SVR) with negative HCV-RNA 24 weeks after the completion of HCV therapy, whereas an early treatment response (EVR; reduction of HCV-RNA of >2 log 10 at week 12) was achieved in only 2 of the 7 (28%) patients. The remaining 2 patients (28%), who had received anti-HCV therapy, were null responders.

## 4. Discussion

Liver transplantation of HIV-infected patients is now considered a reasonable option in the HAART era. Multiple studies have reported promising results of HAART-treated HIV-infected patients with maximally suppressed viral loads, stable CD4 counts, and no significant increase in opportunistic infections after OLT [7, 12–15]. Here, we summarize the results of liver transplantation in HIV-positive patients from Germany. The overall mortality of liver-transplanted HIV-positive patients in Germany was 41% (13/32). These results are comparable to the 36% mortality rate reported by Spanish transplant centres [8]; the mortality rate in HIV-infected patients transplanted in the UK was 30% [9]. Thus, outcomes of liver transplantation in Germany also support the concept that liver transplantation should be offered to selected HIV-positive patients. 

HIV monoinfection or opportunistic infections alone did not seem to be significant risk factors for survival after transplantation in the German patients. Both, in patients from Germany and those reported from other countries, bacterial infections and sepsis had been the leading causes of mortality after liver transplantation, accounting for about 50% of deaths in the patients from Germany, particularly early after transplantation. Of note, 10 of the 13 German patients who had died after liver transplantation also had HCV/HIV coinfection. Current data indicate that HCV co-infection in HIV-positive patients is associated with poor survival after OLT [7–10, 14–16] and that HCV coinfection has a high rate of severe and opportunistic infections [17]. The estimated 5-year survival probabilities for 81 Spanish HIV/HCV-coinfected patients were 47.9%, for 16 coinfected patients in the UK, 53%, for patients from France, 51%, and for the HIV/HCV-positive patients in Germany, 58%. [8, 9, 18]. Thus, survival in HIV-positive patients after liver transplantation was rather similar across the European countries. In contrast, a US study reported a significantly worse five-year patient survival rate of 33% in HCV/HIV-coinfected patients [19], while in a second study the reported 50% five-year survival rate matched those of the European countries [8]. Nevertheless, in a large retrospective cohort analysis Mindikoglu et al. proposed that HIV/HCV-coinfected patients had a significantly lower 2-year survival probability after OLT (52%) than HIV-negative patients with hepatitis C (79%) or HIV-negative patients without HCV infection (81%). In a smaller group of patients, however, 3-year survival of HIV/HCV-co-infected patients after OLT was reported to be similar to patients with HCV monoinfection [20]. These discrepancies probably reflect differences in the management of HCV infection and underline the importance of an aggressive anti-HCV therapy early after transplantation in HIV/HCV-coinfected patients due to developing fibrosing cholestatic hepatitis [21]. 

Treatment for recurrent HCV seems to have a positive effect on graft survival and mortality [22]. Treatment of hepatitis C infection in HIV coinfected patients must be tailored in an individualized manner to account for the prolonged time necessary for elimination of HCV, since clearance of HCV RNA appears to be slower than in HCV-mono-infected patients [23, 24]. Moreover, Vogel et al. showed that delayed initiation of HAART after transplantation and slow tapering of prednisone after stable immunosuppression seem to exert a positive impact on the course of hepatitis C infection [25]. A beneficial effect of anti-HCV therapy is also suggested in our retrospective study, since SVR was achieved in 72% of the HIV/HCV-coinfected patients receiving HCV treatment after OLT. This remarkable outcome of anti-HCV therapy may also reflect meticulous selection of suitable patients for liver transplantation, since a rather poor SVR of 31% was reported for interferon plus ribavirin therapy in a large Spanish cohort of 711 HIV/HCV- infected patients [26]. Nevertheless HCV coinfection still pivotally contributed to the mortality of HIV-positive patients after liver transplantation in the German HIV-positive patients.

Unlike HCV coinfection, HIV-infected patients with HBV seem to have better outcomes after OLT [27] when prophylaxis of HBV reinfection of the graft is correctly provided [12, 27]. This concept is also supported by the German transplant experience in HIV-positive patients, because only a single patient had recurrence of HBV infection under HBV prophylaxis. Of note, survival between HIV/HBV-coinfected patients and patients with HBV monoinfection does not seem to differ after liver transplantation [7, 24]. The King's College Hospital group reported even a survival rate of 100% at 5 years [28]. The 5-year survival rate for HIV/HBV-coinfected patients was 80% in the German patients, and none of the HIV/HBV-coinfected patients developed clinically relevant HBV-related liver disease after OLT. Recurrence of HBV infection in the single patient with hepatitis B recurrence was associated with a regimen not comprising HBs hyperimmunoglobulin, suggesting that prophylaxis of HBV recurrence should comprise both HBs hyperimmunoglobulin and antiviral drugs with anti-HBV activity [27, 29, 30]. Despite successful prevention of recurrent hepatitis B, 50% of the patients on such a combined regimen had detectable low-level HBV viremia in a study but nevertheless achieved 85% patient survival over a followup of 4 years [30]. 

Drugs given as part of HAART as well as immunosuppressive drugs strongly interact with drug-metabolizing enzymes, for example, the cytochrome P450 system. Thus, complex interactions between immunosuppressive regimens and antiretroviral therapy present a particular challenge for the treating physician; they must consider the optimal time of HAART initiation, the drug-drug interactions between HAART and immunosuppressive drugs, as well as control of posttransplant disease recurrence. Starting HAART immediately after OLT may exert a negative effect on mortality, especially in HIV/HCV co-infection, because drug-related liver toxicity may be easily confused with other causes of graft dysfunction in the early posttransplant period [31]. Also 4 of the 10 HIV/HCV-coinfected patients in Germany, who had died, had an early start of HAART in the first 4 days after OLT. The selection of drugs for immunosuppression and antiretroviral therapy is a further critical factor. In the early attempts of liver transplantation in HIV-positive patients with recurrent hepatitis C, dose adjustments of calcineurin inhibitors were not performed; this may have resulted in excessive immunosuppression and death within the first 2 years after OLT [16].

In selecting drugs for HAART potential hepatotoxicity must be taken into consideration in order to reduce liver-associated mortality [31, 32]; the application of antiretroviral agents with low potential for interactions with other drugs, for example, integrase inhibitors, may reduce posttransplant hepatotoxicity of HAART [33]. On the other hand the use of drugs with high hepatotoxic potential such as didanosine (ddI), stavudine (D4T), or azidothymidine (AZT) should be avoided [34]. Furthermore, ddI use is contraindicated in combination with ribavirin because this combination increases the risk of pancreatitis and mitochondrial toxicity. None of the German patients had received ddI but 4 patients had transiently been treated with D4T. Nevertheless D4T drug toxicity is unlikely to have contributed to the high mortality in this subgroup, since the two patients who succumbed had died either from surgical complications or hepatotoxic chemotherapy for lymphoma. 

## 5. Conclusions

Despite some regional differences in the outcome of transplanted HIV-infected patients in Germany, the overall mortality does not differ from that of other European countries. This favorable outcome with acceptable survival rates justifies the strategy to offer liver transplantation to HIV-infected patients with fulminant hepatic failure and end-stage liver disease. The outcome of this option may be improved with better criteria for patient selection and posttransplant management of recurrent liver disease. Nevertheless, the care for such patients still requires a meticulous balance of complex interacting factors such as the choice of HAART, correct dose adjustments of immunosuppressive drugs, and the optimal timing of therapy.

## Figures and Tables

**Table 1:** Demographic data and clinical characteristics of all 32 HIV-positive patients who received a liver graft between July 1997 and July 2011 in Germany.

Age (median (IQR))	**47 **[**37–52**]
Gender (male/female)	31: 01
Risk of HIV infection (*n*(%))^(a)^
Blood products	13/30 (43%)
Homo/heterosexual	9/30 (30%)
Intravenous drug abuse	6/30 (20%)
Unknown	2/30 (7%)
CD4 count (Cell/*μ*L) (median (IQR))^(a)^	266/*μ*L [197–370]
CD4 count >200 (*n* (%))	22/31 (71%)
CD4 count >100 (*n* (%))	7/31 (23%)
CD4 count <100 (*n* (%))	2/31 (6%)
Plasma HIV RNA (<50 copies/mL) (*n* (%))^(a)^	16/31 (52%)
Antiretroviral therapy before OLT (*n* (%))^(a)^
PI-based regimen	17/31 (55%)
Efavirenz-based regimen	6/31 (19%)
Efavirenz and Kaletra	1/31 (3%)
3 NRTI	4/31 (13%)
No ART	3/31 (10%)
Antiretroviral therapy after OLT (*n* (%))^(a)^
PI-based regimen	17/30 (57%)
Efavirenz-based regimen	3/30 (10%)
Efavirenz and Kaletra	1/30 (3%)
3 NRTI	6/30 (20%)
No ART	3/30 (10%)
Reasons for OLT
HCV coinfection	19 (1 ETOH, 5 HCC)
HCV+HBV coinfection	1 (1 HCC)
HBV coinfection	10 (1 ETOH, 1 HCC)
HBV+HCV+HDV coinfection	1
Budd-Chiari syndrome	1
Child-Pugh score and Meld score at the day of OLT^(a)^
Child-Pugh class A (*n* (%))	5/30 (17%)
Child-Pugh class B (*n* (%))	7/30 (23%)
Child-Pugh class C (*n* (%))	18/30 (60%)
Meld (median (IQR))^(a)^	17 [13–29]
Ascites prior to OLT^(a)^	14/29 (48%)
Hepatic encephalopathy prior to OLT^(a)^	8/29 (28%)
Variceal hemorrhage prior to OLT^(a)^	4/29 (14%)

PI: protease inhibitor; NRTI: nucleoside/nucleotide reverse transcriptase inhibitor; ETOH: alcoholic cirrhosis; HCC: hepatocellular carcinoma; HCV: hepatitis C; HBV: hepatitis B; HDV: hepatitis D.

^(a)^Information was not available for all patients.

**Table 2:** HCC in liver-transplanted HIV-infected patients.

Viral hepatitis	Outcome	HCC findings	
HCV	Death	1 HCC focus^§^	
HCV	Living	2 HCC foci^§^ (max. 1.7 cm)	
HCV	Living	3 HCC foci (max 0.7 cm)	
HBV	Living	Multifocal HCC (max. 2 cm)	
HCV	Death, HCC relapse	Multifocal HCC	
HCV	Death, HCC relapse	n.d.	
HCV/HBV	Living	1 HCC focus^§^ (max 1.2 cm)	

HCC: hepatocellular carcinoma; HCV: hepatitis C; HBV: hepatitis B.

n.d.: no data. ^§^HCC was found incidentally on explant pathology.

**Table 3:** Causes of Death of HIV-infected German patients with a liver graft.

Patient No.	Age (yrs)/gender	Cause of end-stage organ failure	CDC stage/CD4 count before OLT	Initial HAART after OLT	Start of HAART (days after OLT)	HCV or HBV therapy	Followup (Months)	Causes of death
1	58/m	HCV	A3/168/*μ*L	D4T/ 3TC/ABC	53	IFN/RBV	93	Kidney failure

2	37/m	HCV/HBV/HDV	C3/86/*μ*L	AZT/3TC/ABC	2	IFN/RBV 3,5 months after OLT	56	Re-retransplantation for primary graft failure, progressive hepatitis, HCV-positive donor organ, sepsis

3	36/m	HCV	A3/219/*μ*L	3TC/D4T/EFV	4	No HCV therapy	31	Secondary graft failure for chemotherapy for Hodgkin's disease and chronic HCV

4	41/m	HCV	A2/380/*μ*L	TVD/FPV/RTV	75	No HCV therapy	25	Progressive metastasis of HCC

5	52/m	HCV	B1	LPV/EFV	1	No HCV therapy	13	Progressive sceletal metastasis of HCC, abscess after TACE

6	48/f	HCV	A1/621/*μ*L	AZT/3TC/NLF	2	No HCV therapy	10	Secondary graft failure for progressive cholestatic hepatitis

7	45/m	HCV	B2/372/*μ*L	ABC/3TC/EFV	34	IFN/RBV therapy	8	Anastomic leak with massive bleeding, multiorgan failure, sepsis

8	57/m	HBV	A3/202/*μ*L	D4T/ 3TC/ABC	55	HBs-Ig/lamivudin	3	Intrathoracic hemorrhage after placement of a thoracic drain

9	49/m	HBV	B3/196/*μ*L	AZT/ 3TC	29	HBs-Ig/lamivudin	3	Thrombosis of *A. hepatica*, graft failure due to organ rejection, biliary leakage and intra-abdominal abscess, sepsis, multiorgan failure

10	45/m	HCV	C3/193/*μ*L	LPV/SQV	1	No HCV therapy	3	Pseudomonas sepsis, kidney failure after combined liver kidney transplantation

11	57/m	HCV	—/622/*μ*L	no data	no data	No HCV therapy	2	Retransplantation due to primary graft nonfunction and organ rejection, kidney failure

12	48/m	HCV	B2/210/*μ*L	no data	no data	No HCV therapy	1	Sepsis, multiorgan failure

13	33/m	Budd-Chiari-Syndrome	B2/595/*μ*L	none	no data	no data	1	Sepsis

3TC: lamivudine, ABC: abacavir, D4T: stavudine, EFV: efavirenz, AZT: azidothymidine, NLF: nelfinavir, LPV: lopinavir, SQV: saquinavir, TVD: truvada, FPV: fosamprenavir, RTV: ritonavir, TACE: transarterial chemoembolisation, NTX: kidney transplantation, IFN: interferon, RBV: ribavirin, HBs-Ig: HBs hyperimmunoglobulin, HCV: hepatitis C virus, HBV: hepatitis B virus, HDV: hepatitis D virus, m: male, f: female.

**Table 4:** Recurrence and prevention of hepatitis B in HIV-positive patients after OLT.

Viral hepatitis	HBV therapy	HBV virus
HBV	3TC, HBs-Ig	HBsAg negative
HBV	3TC, HBs-Ig	HBsAg negative
HBV	3TC, HBs-Ig	HBsAg negative
HBV	TDF, HBs-Ig	HBsAg negative
HBV	TDF, HBs-Ig	HBsAg negative
HBV	3TC	HBsAg negative
HBV/HCV/HDV	3TC	HBsAg negative
HBV	3TC, entecavir	HBsAg negative
HBV	TDF, 3TC	low viremia
HBV	TDF	HBsAg negative
HBV/HCV	TDF, HBs-Ig	HBsAg negative
HBV	3TC	HBsAg negative

3TC: lamivudine, TDF: tenofovir, HBs-Ig: HBs hyperimmunoglobulin, HCV: hepatitis C virus, HBV: hepatitis B virus, HDV: hepatitis D virus.

**Table 5:** Recurrence of hepatitis C in HIV-infected patients and outcome after OLT.

Viral hepatitis	HCV genotype	HCV therapy	Nonresponse	Survival, followup (months)
HCV	2a/2c	IFN/RBV/amantadine	SVR	Living, 114
HCV	3a	IFN/RBV	SVR	Death, 93
HCV	1a	IFN/RBV/amantadine	EVR/SVR	Living, 89
HCV	1a	IFN/RBV/amantadine	EVR/SVR	Living, 82
HCV	1a	IFN/RBV	SVR	Living, 66
HCV/HBV/HDV	1a	IFN/RBV	Nonresponse	Death, 56
HCV	1a/b	IFN/RBV	Nonresponse	Death, 8
HCV	n.a.	No	/	Death, 31
HCV	3a	No	/	Death, 25
HCV	n.a.	No	/	Death, 13
HCV	n.a.	No	/	Death, 10
HCV	1a	No	/	Death, 3
HCV	n.a.	No	/	Death, 2
HCV	n.a.	No	/	Death,1
HCV	n.a.	No	/	Living, 74
HCV	1b	n.a.	n.a.	Living, 32
HCV	1b	n.a.	n.a.	Living, 67
HCV	3a	No	/	Living, 39
HCV/HBV	1	No	/	Living, 18
HCV	1a	No	/	Living, 44
HCV	n.a.	No	Spontaneous viral clearance	Living, 54

IFN: interferon, RBV: ribavirin, EVR: early virological response, SVR: sustained virological response, HCV: hepatitis C virus, HBV: hepatitis B virus, HDV: hepatitis D virus.
